# Cysteine-rich protein 1 (CRP1) regulates actin filament bundling

**DOI:** 10.1186/1471-2121-6-45

**Published:** 2005-12-08

**Authors:** Thuan C Tran, CoreyAyne Singleton, Tamara S Fraley, Jeffrey A Greenwood

**Affiliations:** 1 Department of Biochemistry and Biophysics, Oregon State University, Corvallis, Oregon 97331, USA

## Abstract

**Background:**

Cysteine-rich protein 1 (CRP1) is a LIM domain containing protein localized to the nucleus and the actin cytoskeleton. CRP1 has been demonstrated to bind the actin-bundling protein α-actinin and proposed to modulate the actin cytoskeleton; however, specific regulatory mechanisms have not been identified.

**Results:**

CRP1 expression increased actin bundling in rat embryonic fibroblasts. Although CRP1 did not affect the bundling activity of α-actinin, CRP1 was found to stabilize the interaction of α-actinin with actin bundles and to directly bundle actin microfilaments. Using confocal and photobleaching fluorescence resonance energy transfer (FRET) microscopy, we demonstrate that there are two populations of CRP1 localized along actin stress fibers, one associated through interaction with α-actinin and one that appears to bind the actin filaments directly. Consistent with a role in regulating actin filament cross-linking, CRP1 also localized to the membrane ruffles of spreading and PDGF treated fibroblasts.

**Conclusion:**

CRP1 regulates actin filament bundling by directly cross-linking actin filaments and stabilizing the interaction of α-actinin with actin filament bundles.

## Background

Stress fibers are bundles of actin microfilaments formed in cells following integrin-mediated attachment and spreading [1]. Regulation of these contractile fibers is critical for cell adhesion and motility. The microfilaments within stress fibers are held together by specialized bundling proteins, such as myosin and α-actinin, which can interact simultaneously with two actin filaments. These classical actin-bundling proteins have been studied extensively leading to a basic understanding of their interaction with actin filaments and regulation of stress fibers. One important discovery was the periodic and alternating association of myosin and α-actinin which is clearly visualized as a beaded pattern along stress fibers in cells stained for immunofluoresence microscopy [2]. Although it is not understood how this alternating association of myosin and α-actinin with the microfilaments is regulated, it is critical for the contractility of the stress fiber. In addition to myosin and α-actinin, actin stress fibers are decorated with numerous other proteins, associated either directly or through interaction with other actin-binding proteins. Determining the function of these ancillary proteins is important for understanding the regulation of stress fibers.

Modulation of the actin cytoskeleton by LIM domain proteins is an active and growing field of research [3]. LIM domains are cysteine-rich sequences of 50–60 amino acid residues that contain two tandem zinc fingers [4]. Several LIM proteins have been demonstrated to interact with and/or regulate α-actinin. Vallenius et al. [5] have reported that reversion-induced LIM (RIL) protein associates with α-actinin increasing its binding to actin filaments *in vitro *and may alter actin stress fibers in various cell types. Four and a half LIM domain protein 3 (FHL3) has been demonstrated to disrupt actin stress fibers in C2C12 myoblasts presumably by binding to actin filaments and inhibiting α-actinin bundling [6]. In addition, ENH [7], ALP [8], Cypher [9], CLIM1 [10], CLP-36 [11,12], zyxin [13], and the cysteine-rich protein (CRP) family [14,15] have also been demonstrated to interact with α-actinin, although it is not clear if these proteins influence α-actinin function.

The CRP family, which includes CRP1, CRP2, CRP3, and the thymus LIM protein (TLP), is a subgroup of LIM domain proteins containing two LIM domains linked to short glycine-rich repeats [16]. CRPs are highly conserved between species with CRP1 from human, chicken, and quail having an amino acid sequence identity greater than 90% [17]. Although the CRPs have different patterns of expression, evidence suggests that they are functionally similar [14,16]. CRPs are important for cell differentiation presumably by modulating protein-protein interactions involved in transcriptional regulation [16,18]. Outside of the nucleus, CRPs clearly localize to focal adhesions and the actin cytoskeleton, and have been postulated to play a role in controlling these structures [16].

CRP1, CRP2, and CRP3, have all been shown to bind α-actinin [14,15]. Interaction between the two proteins was first reported for CRP1 using affinity chromatography, solution and solid-phase binding assays, and co-immunoprecipitation from cell lysates [15]. A follow up study demonstrated that α-actinin could interact equally with CRP1, CRP2, and CRP3 [14]. In addition, it was determined that the CRPs were interacting with the actin-binding domain of α-actinin [15] and the α-actinin-binding site was mapped to amino acid residues 62–79 of human CRP1 [19]. Based on the binding assays and co-localization within the cell, it was proposed that the interaction of CRP with α-actinin was responsible, in part, for its localization to the actin cytoskeleton [14,15]. Recently, Grubinger and Gimona [20] demonstrated that CRP2 binds directly to actin filaments and suggested that CRP2 does not interact with α-actinin. We have found that there are two populations of CRP1 associated with actin stress fibers, one interacting with α-actinin and one that appears to interact directly with the actin filaments. Although, CRPs have been shown to bind to α-actinin and actin filaments, it is not clear how these proteins regulate the actin cytoskeleton. In this study, we show that CRP1 regulates actin filament bundling by directly cross-linking actin filaments and stabilizing the interaction of α-actinin with actin filament bundles.

## Results

### Expression of CRP1 increases F-actin bundling in REFs

**Figure 1:**
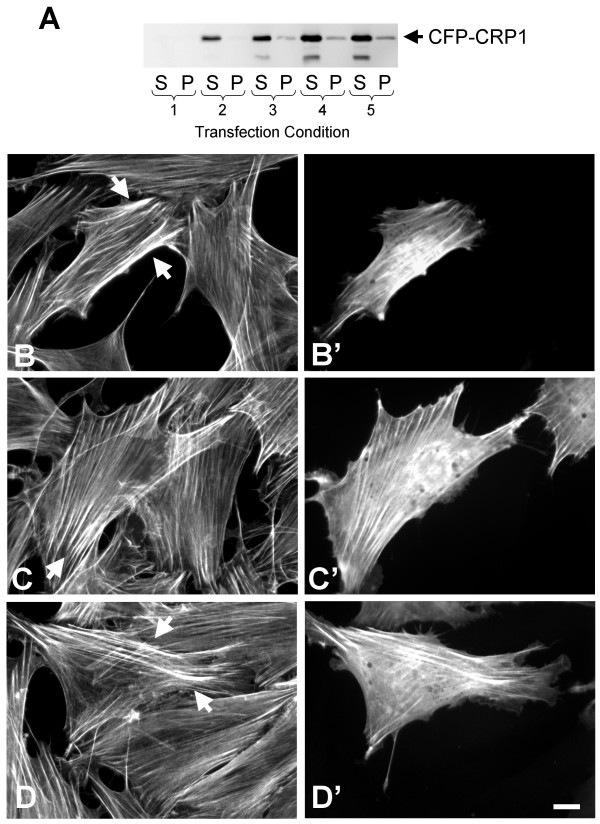
**Expression of CRP1 increases the bundling of cellular actin filaments**. REFs transfected with pECFP-CRP1 as described in Experimental Procedures were lysed and the Triton X-100 soluble and insoluble fractions separated for analysis. The transfection conditions were: **1) **6 μL FuGENE, no DNA; **2) **3 μL FuGENE, 0.5 μg DNA; **3) **3 μL FuGENE, 1.0 μg DNA; **4) **6 μL FuGENE, 1.0 μg DNA; and **5) **6 μL FuGENE, 2.0 μg DNA. Immunoblot shows the expression of CFP-CRP1 in each fraction (**A**). REFs transfected with pECFP-CRP1 using transfection condition 4 were fixed and prepared for fluorescence microscopy. Rhodamine-phalloidin staining of F-actin (**A, B, C**) shows that the cells expressing CFP-CRP1 (**A', B', C'**) have enlarged stress fibers (see arrows). Results are representative of 4 separate experiments. Bar = 10 μm.

To determine the influence of CRP1 on the actin cytoskeleton, rat embryonic fibroblasts (REFs) were transfected with increasing concentrations of DNA encoding CFP-CRP1 resulting in increasing levels of expression of the fluorescent fusion protein (Fig. 1A). Expression of CFP-CRP1 was observed in approximately 15% of the cells with levels ranging from 2–4 fold that of endogenous CRP1 (data not shown). Consistent with immunostaining for endogenous CRP1 (see Additional files 1 and 2) and previous reports [14-16,21,22], CFP-CRP1 was localized diffusely in the cytoplasm, along the actin cytoskeleton, and in the nucleus of some cells (~10%). These results suggest that the CFP tag does not significantly affect the function of the CRP1 protein. Although the morphology of the REFs was unaffected, expression of CFP-CRP1 appeared to increase the bundling of actin microfilaments (Fig. 1, see arrows). If the expression of CFP-CRP1 was resulting in increased actin filament bundling, then CFP-CRP1 would be expected to redistribute to the Triton X-100 insoluble cytoskeletal fraction. As shown in Fig. 1A, insoluble CFP-CRP1 increased with expression. In addition, an immunoreactive band migrating below CFP-CRP1 was recognized, presumably a proteolytic fragment. The distribution of actin and α-actinin in the Triton X-100 soluble and insoluble fractions were also examined. However, with a transfection efficiency of only 15%, a significant change in the insolubility of actin and α-actinin was not detected (data not shown).

### CRP1 bundles F-actin independently of α-actinin

**Figure 2:**
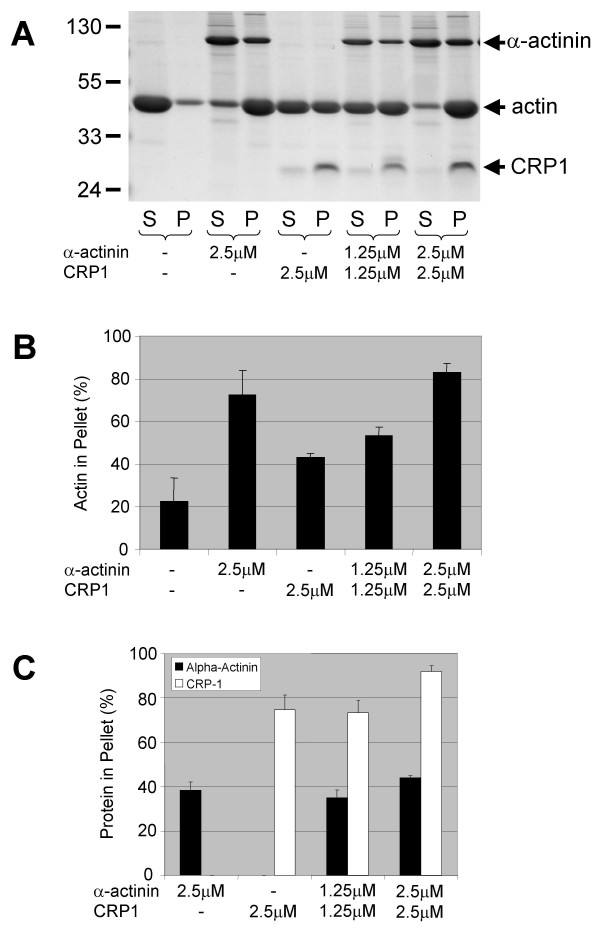
**CRP1 does not influence the bundling activity of α-actinin**. α-Actinin and CRP1 were preincubated for 15 min at room temperature. Actin filaments were then added, incubated for 30 min at room temperature, and centrifuged at 10,000 × g. Proteins from the supernatant (S) and the pellet (P) were separated by electrophoresis and detected by Gelcode Blue staining (**A**). The percentage of total actin (**B**) and α-actinin and CRP1 (**C**) in the pellet was quantified as described in Experimental Procedures. n = 4 ± SEM.

**Figure 3:**
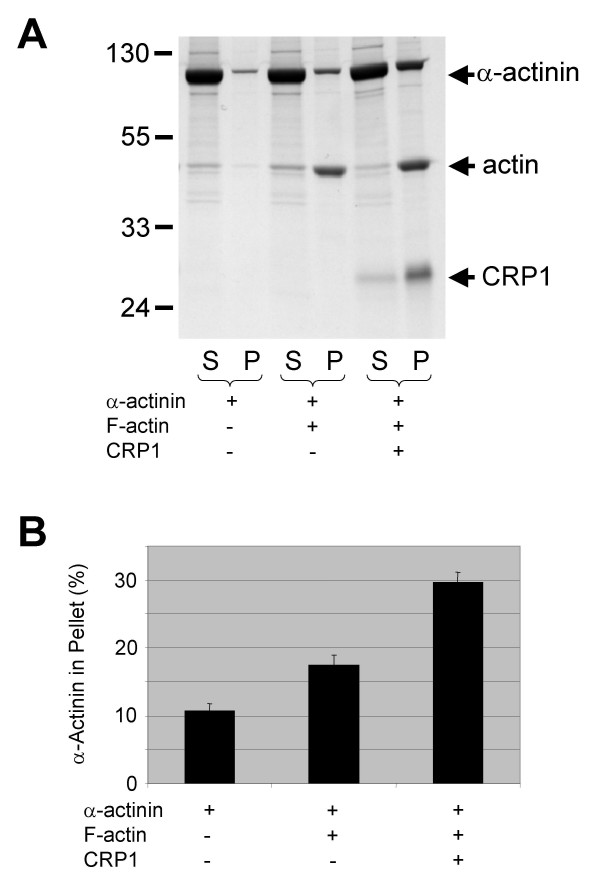
**CRP1 stabilizes the interaction of α-actinin with actin bundles**. Actin filaments were bundled by incubation with α-actinin for 30 min. at room temperature. CRP1 was then added, incubated for an addition 30 min., and centrifuged at 10,000 × g. Proteins from the supernatant (S) and the pellet (P) were separated by electrophoresis and detected by Gelcode Blue staining (**A**). The percentage of total α-actinin in the pellet was quantified as described in Experimental Procedures (**B**). n = 3 ± SEM.

Since CRP1 has been demonstrated to bind to α-actinin [15], we proposed that CRP1 was regulating actin filament bundling by increasing α-actinin bundling activity. In order to test this hypothesis, α-actinin bundling activity was examined in the absence and presence of CRP1. For these experiments (Fig. 2), actin filament bundling was determined using a sedimentation assay with 2.5 μM α-actinin alone, 2.5 μM CRP1 alone, 1.25 μM α-actinin + 1.25 μM CRP1 (2.5 μM total protein), and 2.5 μM α-actinin + 2.5 μM CRP1 (5.0 μM total protein). Although, CRP1 did not appear to influence the bundling activity of α-actinin, we did find that when CRP1 was added to α-actinin bundled actin filaments, a 2-fold increase in α-actinin was observed in the bundles (Fig. 3). These results suggest that CRP1 was stabilizing the interaction of α-actinin with the actin filaments in the bundles.

**Figure 4:**
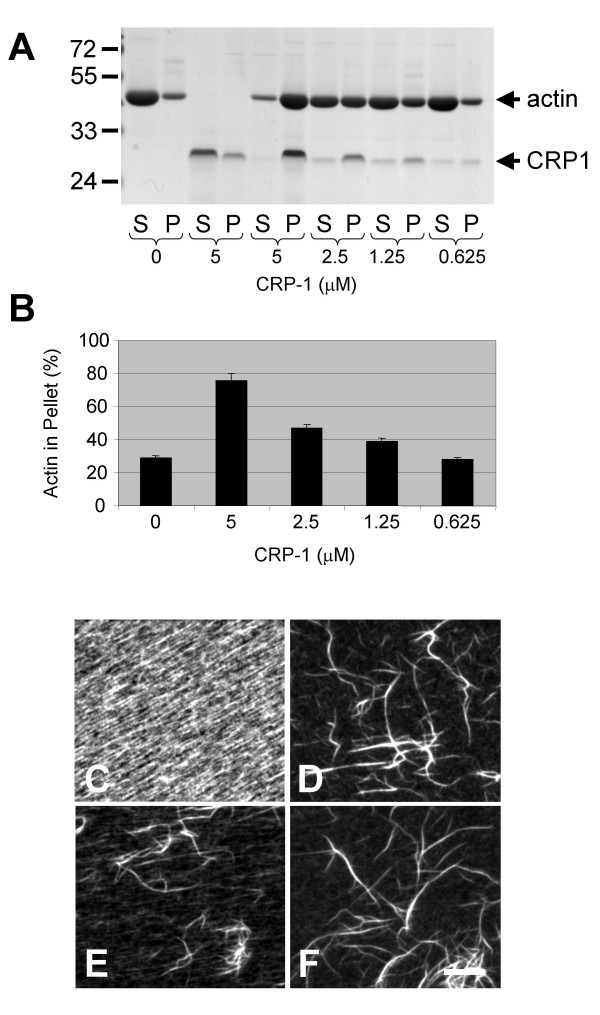
**CRP1 bundles F-actin independently**. The indicated concentration of CRP1 was incubated with actin filaments for 30 min at room temperature and centrifuged at 10,000 × g. Proteins from the supernatant (S) and the pellet (P) were separated by electrophoresis and detected by Gelcode Blue staining (**A**). The percentage of total actin in the pellet was quantified as described in Experimental Procedures (**B**). n = 4 ± SEM. Bundling reactions were also carried out on glass coverslips, fixed, and stained with rhodamine-phalloidin to allow visualization of the actin filaments bundles: (**C**) actin filaments, (**D**) actin filaments + 2.5 μM α-actinin, (**E**) actin filaments + 5.0 μM CRP1, and (**F**) actin filaments + 2.5 μM α-actinin + 2.5 μM CRP1. Results are representative of 4 separate experiments. Bar = 10 μm.

We also found that CRP1 alone could bundle actin filaments in a concentration dependent manner (Figs. 2 and 4). Furthermore, CRP1 bound to the actin filaments more efficiently than α-actinin, 75–90% compared to 35–45%, under all of the experimental conditions (Fig. 2C). To confirm that CRP1 was bundling the actin filaments, the bundling assay was carried out on glass coverslips and the proteins fixed with 3% formaldehyde followed by staining with rhodamine-phalloidin (Fig. 4C–F). Images of the samples clearly show that CRP1 induced the formation of actin filament bundles similar to that of α-actinin.

### Two populations of CRP1 are associated with actin stress fibers

**Figure 5:**
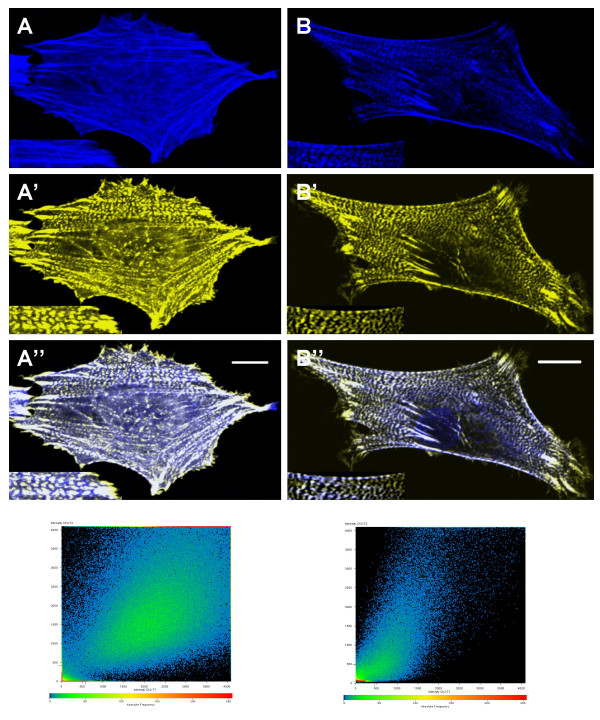
**Two populations of CRP1 are localized along stress fibers**. REFs expressing CFP-CRP1 and YFP-α-actinin were fixed with 3% formaldehyde in PBS (**A**) or Triton X-100 buffer (**B**) and prepared for confocal microscopy as described in Experimental Procedures. Images of CFP-CRP1 (**A **and **B**) and YFP-α-actinin (**A' **and **B'**) localization are shown. A zoomed region is shown in the lower left corner of each image. Merged images are shown in **A" **and **B"**. Results are representative of 4 separate experiments. Bar = 10 μm.

Previous reports have suggested that CRP1 is localized to actin filaments through interaction with α-actinin [14,15]. Since the above results demonstrated that CRP1 could directly bind to and bundle actin filaments, we asked if CRP1 was actually binding to α-actinin along actin stress fibers within the cell. REFs were co-transfected with DNA encoding CFP-CRP1 and YFP-α-actinin. As in previous experiments, approximately 15% of the cells expressed the tagged proteins with greater than 90% of these cells expressing both proteins. In cells fixed with 3% formaldehyde in PBS using standard protocols, YFP-actinin was observed in its classical beaded pattern along actin stress fibers and within focal adhesions (Fig. 5A). CFP-CRP1 was also observed in focal adhesions and along actin stress fibers; however localization along the stress fibers was continuous and not beaded like that of α-actinin (Fig. 5A). Cells were also fixed with 3% formaldehyde in Triton X-100 buffer in order to remove the soluble cytoplasmic proteins and proteins weakly associated with the cytoskeleton. Using this fixation protocol, CFP-CRP1 was observed to co-localize explicitly with YFP-α-actinin along actin stress fibers and in focal adhesions (Fig. 5B). Scatter plots are shown in Fig. 5 to represent localization of the two proteins in the images. For the unextracted cell, the distribution of fluorescence intensity for the two tags is diffuse as a result of the areas in the cell where the two proteins are not co-localized, with a correlation coefficient of R = 0.4. For the extracted cell, the co-localized populations of the two proteins are represented by the characteristic comet shaped scatter plot with a correlation coefficient of R = 0.7.

**Figure 6:**
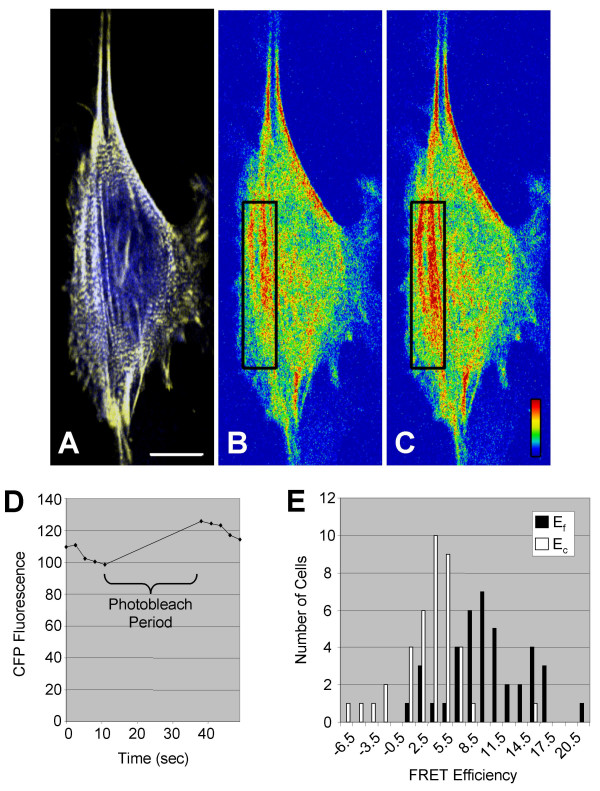
**CRP1 binds α-actinin along actin stress fibers**. REFs were co-transfected with pECFP-CRP1 and pEYFP-α-actinin and FRET microscopy carried out as described in Experimental Procedures. (**A**) Overlay image of cell expressing CFP-CRP1 and YFP-α-actinin showing the ROI that was photobleached during the FRET experiment. A color scaled image of the CFP fluorescence of the cell prior to (**B**) and immediately following (**C**) the photobleaching period. Red represents a high signal and blue, a low signal. Bar = 10 μm. (**D**) The intensity of the CFP fluorescence within the ROI was quantified for each image captured during the experiment. (**E**) The distribution of FRET efficiencies for the photobleached ROIs, E_f_, and control non-photobleached ROIs, E_c_, were plotted on the bar graph.

Although we had clearly identified a population of CRP1 that co-localized with α-actinin, this did not prove that the two proteins were interacting directly with each other. An established method for determining the interaction between two localized proteins in a cell involves fluorescence resonance energy transfer (FRET) microscopy. In these experiments, we followed the method for photobleaching FRET (pbFRET) described by Karpova et al. [23] using REFs co-expressing CFP-CRP1 (donor) and YFP-α-actinin (acceptor). For this method, the FRET efficiency is determined by measuring the dequenching of the donor emission after selective photobleaching of the acceptor [23,24]. In cells fixed with 3% formaldehyde in Triton X-100 buffer, we consistently observed an increase in CFP emission along actin stress fibers following photobleaching of the YFP (Fig. 6A–D). As a control, the CFP emission on a similar region which was not photobleached was also measured. The histogram in Fig. 6E shows that the distributions of the FRET efficiencies for the photobleached regions (E_f_) was shifted positively compared to the unbleached control regions (E_c_) with the mean E_f _significantly different from the control E_c _(9.6 ± 0.74 vs. 2.9 ± 0.61, p < 0.05). For comparison, the FRET efficiency for a CFP-YFP fusion protein was reported as 7.96 ± 0.38 for the photobleached regions compared to 2.21 ± 0.26 for the unbleached control regions [23].

### CRP1 localizes to dynamic actin structures

**Figure 7:**
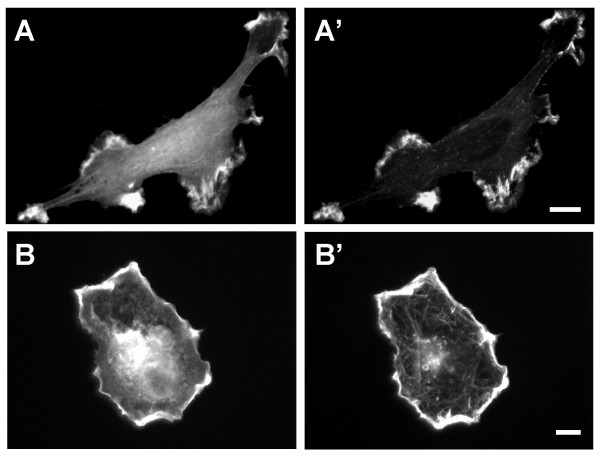
**CRP1 localizes to dynamic actin structures**. REFs expressing CFP-CRP1 (**A **and **B**) and YFP-α-actinin (**A' **and **B'**) were stimulated with 30 ng/ml PDGF for 30 min or plated on fibronectin for 30 min as previously described [25], fixed with 3% formaldehyde in Triton X-100 buffer, and prepared for microscopy as described in Experimental Procedures. Results are representative of 3 separate experiments. Bar = 10 μm.

α-Actinin is not only localized to stress fibers and focal adhesions, but also to more dynamic actin structures such as the membrane ruffles of spreading and PDGF treated cells. Thus, if CRP1 cross-links actin filaments and stabilizes the interaction of α-actinin with actin filaments, localization to membrane ruffles would also be expected. Previously, we have shown that platelet-derived growth factor (PDGF) stimulates the relocation of α-actinin to membrane ruffles [25]. In order to determine if CRP1 would relocalize with α-actinin, REFs co-expressing YFP-α-actinin and CFP-CRP1 were stimulated with PDGF for 30 min followed by fixation in Triton X-100 buffer. Images of the two proteins clearly show that CFP-CRP1 is co-localized with α-actinin in the membrane ruffles of PDGF treated cells (Fig. 7A). Experiments were also carried out with co-expressing cells showing the co-localization of CRP1 and α-actinin to the membrane ruffles of REFs during cell spreading (Fig. 7B).

## Discussion

CRP1 is a LIM domain containing protein which has been demonstrated to localize in the nucleus, the cytoplasm, along actin filaments, and in focal adhesions. Recent studies have shown that the nuclear population of CRPs mediates protein-protein interactions regulating transcription and differentiation[18]. Although CRPs have been found to bind to α-actinin, zyxin, and actin filaments, little is known about how CRPs regulate the actin cytoskeleton. In this study, we examined the regulation of actin filaments by CRP1 *in vitro *and in cultured cells.

Expression of CRP1 in fibroblasts resulted in increased actin filament bundling (Fig. 1). In addition, we found that CRP1 directly binds to and bundles actin filaments (Fig. 2 and 4). CRP2 has recently been reported to bind actin filaments [20]; however, this is the first study to show that any CRP can bundle actin filaments.

After observing an increase in actin filament bundling in the cells expressing CFP-CRP1, we expected to find that CRP1 was enhancing the bundling activity of α-actinin. However, *in vitro *bundling studies clearly demonstrated that CRP1 had no influence on the ability of α-actinin to bundle actin filaments (Fig. 3). Furthermore, these results showed that CRP1 and α-actinin do not compete for binding to actin filaments and therefore must bind at different sites. Interestingly, approximately twice as much CRP1 protein pellets with the actin filament bundles compared to α-actinin. The significance of these findings is not clear, but may reflect differences in the mechanisms by which CRP1 and α-actinin bind and bundle actin filaments. Further studies are necessary to determine how CRP1 is bundling actin filaments.

Although α-actinin had previously been demonstrated to bind and presumably localize CRP1 to actin filaments [14,15], the new evidence that CRP1 could directly bind and bundle actin filaments prompted further investigation. Detergent extraction of cells has long been used to improve examination of the cytoskeleton. Previously, we demonstrated that Triton X-100 extraction removes soluble cytoplasmic proteins leaving behind an intact cytoskeleton with associated adhesion and matrix proteins[25]. Extraction with Triton X-100 has also been used to separate and define adhesion and cytoskeletal proteins based on stable association with the actin cytoskeleton [26]. Triton X-100 extraction of fibroblasts co-expressing CFP-CRP1 and YFP-α-actinin allowed us to differentiate two populations of CRP1 along actin stress fibers (Fig. 5). The Triton X-100 resistant population of CRP1 co-localized with α-actinin along stress fibers, whereas the less stable Triton X-100 susceptible population appeared to represent CRP1 which was directly associated with actin filaments. Furthermore, confocal microscopy demonstrated that CFP-CRP1 and YFP-α-actinin were in close enough proximity for FRET between the CFP and YFP (Fig. 6). Since the tags need to be within ~50Å for efficient FRET to occur [24], the results indicate that CRP1 and α-actinin are bound to each other along actin stress fibers.

## Conclusion

Evidence from this and other studies suggests that the different populations of CRP1 mediate protein-protein interactions unique to each cellular compartment. Nuclear CRP1 regulates interactions between transcription factors[18]. Cytoplasmic CRP1 modulates the actin cytoskeleton by two mechanisms: 1) stabilizing α-actinin interaction with actin bundles and 2) cross-linking actin filaments. Further studies are needed to determine how the different populations of CRP1 coordinate to modulate the function of the cell.

## Methods

### Proteins and DNA constructs

α-Actinin was purified from chicken gizzard as previously described [27]. Non-muscle actin (99% pure; 80% β-actin, 20% γ-actin) was polymerized following the manufacturer's protocol (Cytoskeleton, Inc., Denver, CO). The plasmid containing chicken CRP1 cloned into the EcoRI site of pBlueScript II KS (pBSIIKS, Stratagene) was generously provided by Mary C. Beckerle (Univ. of Utah) [15]. The EcoRI insert of CRP1-pBSIIKS was sub-cloned into the enhanced cyan fluorescent fusion protein vector pECFP-C1 (BD Biosciences) and the BamHI-HindIII insert sub-cloned into pProEx HTb (Invitrogen). Nucleotide sequences were confirmed by sequence analysis. His-tagged CRP1 protein was expressed in BL21 bacteria and purified using Ni-NTA resin (Qiagen) following procedures described by the manufacturer. The his tag was cleaved from CRP1 while still bound to the resin using recombinant TEV protease (Invitrogen) following the manufacturer's protocol. The untagged CRP1 protein was concentrated and buffer-exchanged (10 mM HEPES, pH 7.0, 50 mM NaCl, 1 mM EDTA) in a centrifugal filter device (Amicon). YFP-α-actinin was constructed by subcloning the α-actinin gene [28] into the HindIII restriction site of the enhanced yellow fluorescent fusion protein vector pEYFP-N1 (BD Biosciences).

### F-actin bundling assays

The bundling of F-actin was determined by sedimentation assays as previously described [28,29]. F-actin (10.4 μM) was incubated with the indicated concentration of CRP1 or α-actinin in bundling buffer (10 mM HEPES, pH 7.0, 50 mM NaCl, 1 mM EDTA) for 30 min at room temperature and centrifuged at 10,000 × g for 30 min. The supernatant and pellet were separated and the proteins analyzed by electrophoresis. Proteins were detected by Gelcode Blue (Pierce) staining and quantified using a KODAK ImageStation 440CF.

F-actin bundles were visualized by fluorescence microscopy following modification of previously described procedures [30,31]. Briefly, 50 μl of assay solution was incubated on a glass coverslip inside a 12-well tissue culture dish. After 30 min, the proteins were fixed by adding 3% formaldehyde in phosphate buffered saline for an additional 30 min. Coverslips were then stained and processed for microscopy as described previously[25].

### Cell culture and fluorescence microscopy

Rat embryonic fibroblasts (REFs) were cultured as described previously [25]. Cells were transfected with pECFP-CRP1 and pEYFP-α-actinin using FuGENE 6 (Roche) following manufacturer's protocols. The expression curve of CFP-CRP1 was carried out by varying the ratio of FuGENE to DNA in a final volume of 100 μL serum-free media. The transfection conditions were: 6 μL FuGENE, no DNA; 3 μL FuGENE, 0.5 μg DNA; 3 μL FuGENE, 1.0 μg DNA; 6 μL FuGENE, 1.0 μg DNA; and 6 μL FuGENE, 2.0 μg DNA. Twenty-four hours after transfection, cells were prepared for fluorescence microscopy or scraped into ice-cold lysis buffer (10 mM Tris, pH 7.4, 150 mM NaCl, 1 mM EGTA, 1 mM EDTA, 2 mM Na_3_VO_4_, 1% Triton X-100, 0.5% NP-40, 30 mM sodium pyrophosphate, 50 mM NaF, 1 μg/ml leupeptin, and 1 μg/ml aprotinin) as described previously [25]. The lysates were centrifuged at 10,000 × g for 10 min at 4°C, protein from the supernatant and pellet separated by electrophoresis, and immunoblotted with anti-GFP (Santa Cruz), anti-α-actinin (Chemicon), or anti-actin (Sigma). Proteins were detected by enhanced chemiluminence (Pierce) and quantified using a KODAK ImageStation 440CF. For fluorescence microscopy, cells were fixed for 30 min at room temperature with 3% formaldehyde (Tousimis) in PBS or in Triton X-100 buffer (20 mM Tris, pH 7.4, 50 mM NaCl, 1 mM EGTA, 5 mM EDTA, 100 μM Na_3_VO_4_, 50 mM sodium pyrophosphate, 1 μg/mL leupeptin, 1 μg/mL aprotinin, and 0.5 % Triton X-100). Digital images were captured using a Zeiss axiovert 100S microscope equipped with a Photometrics CoolSNAP HQ CCD camera controlled by MetaMorph software. Co-localization studies were carried out using a Zeiss LSM 510 confocal microscope. The scatter plots and correlation coefficients were determined using Zeiss Physiology Software v3.2.

### Fluorescence resonance energy transfer (FRET) microscopy

REFs were co-transfected with pECFP-CRP1 and pEYFP-α-actinin, cultured for an additional 24 hrs, fixed using 3% formaldehyde in Triton X-100 buffer, and prepared for confocal microscopy as described above. The FRET assays were carried out following the procedure described by Karpova et al. [23]. Briefly, cells were imaged with a Zeiss LSM 510 confocal microscope operated by Zeiss Physiology Software v3.2 using a 63 × 1.3 NA Zeiss oil immersion lens. The microscopy system was configured in multitracking mode to excite the CFP with a 458 nm and YFP with a 514 nm laser line. A region of interest (ROI) containing actin stress fibers was selected for photobleaching. Using the time series function, 5 images of the cell were collected followed by selective photobleaching of the YFP within the ROI with the 514 nm laser line (typically, 150 iterations at 100% laser power was sufficient), and then the collection of 5 additional images. The FRET efficiency was calculated as a percentage using the following formula E = 100 × (I_postbleach _– I_prebleach_)/I_prebleach_, where I is the intensity of CFP fluorescence within the ROI. As a control, ROIs were selected from non-bleached regions of the cell.

## List of abbreviations

The abbreviations used are: RIL, reversion-induced LIM; FHL3, Four and a half LIM domain protein 3; ENH, enigma homologue protein; ALP, actinin-associated LIM protein; CLIM, 36kDa carboxyl terminal LIM domain protein; CRP, cysteine-rich protein; F-actin, filamentous actin; PDGF, platelet-derived growth factor; CFP, cyan fluorescent protein; YFP, yellow fluorescent protein; REFs, rat embryonic fibroblasts.

## Authors' contributions

TCT carried out the cell and molecular studies. CS carried out the *in vitro *bundling assays and spreading experiments. TSF participated in the confocal microscopy analysis. JAG conceived of the study, and participated in its design and coordination and drafted the manuscript. All authors read and approved the final manuscript.

## Supplementary Material

Additional File 1 Immunostaining for endogenous CRP1. Fluorescence microscopy of REFs stained with antibodies recognizing the C-terminal 17 amino acids residues (PKGFGFGQGAGALVHSE) of rat CRP1. Bar = 10 μm. Click here for file

Additional File 2 Immunostaining for endogenous CRP1. Fluorescence microscopy of REFs stained with antibodies recognizing the C-terminal 17 amino acids residues (PKGFGFGQGAGALVHSE) of rat CRP1. Bar = 10 μm. Click here for file
