# Supplementary figures and images for: Cysteine-rich protein 1 (CRP1) regulates actin filament bundling
Source: BMC Cell Biol. 2005 Dec 8;6:45. doi: 10.1186/1471-2121-6-45 (PMC1318456; doi:10.1186/1471-2121-6-45)

## Slide 1
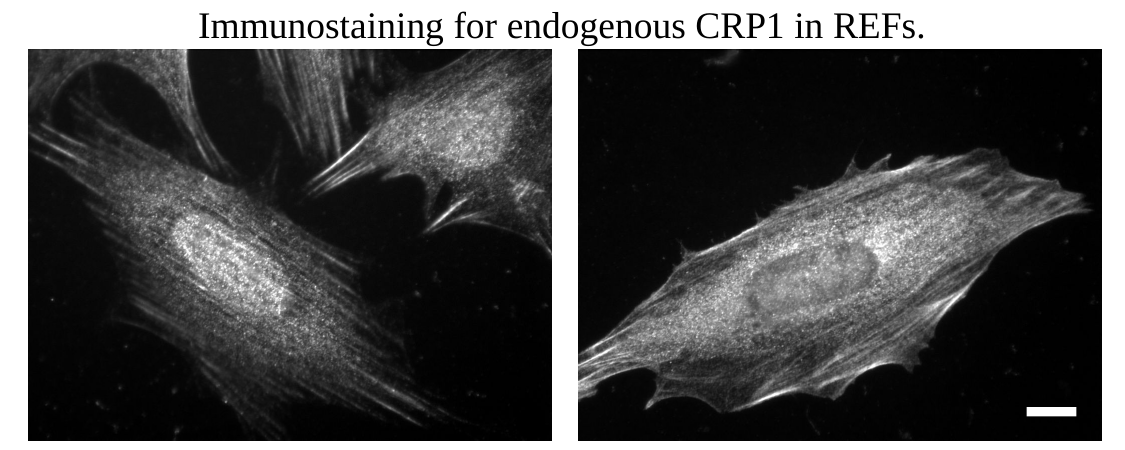

Immunostaining for endogenous CRP1 in REFs.

Supplement: Additional File 1 — Immunostaining for endogenous CRP1. Fluorescence microscopy of REFs stained with antibodies recognizing the C-terminal 17 amino acids residues (PKGFGFGQGAGALVHSE) of rat CRP1. Bar = 10 μm. [file 1471-2121-6-45-S1.ppt]

## Slide 1
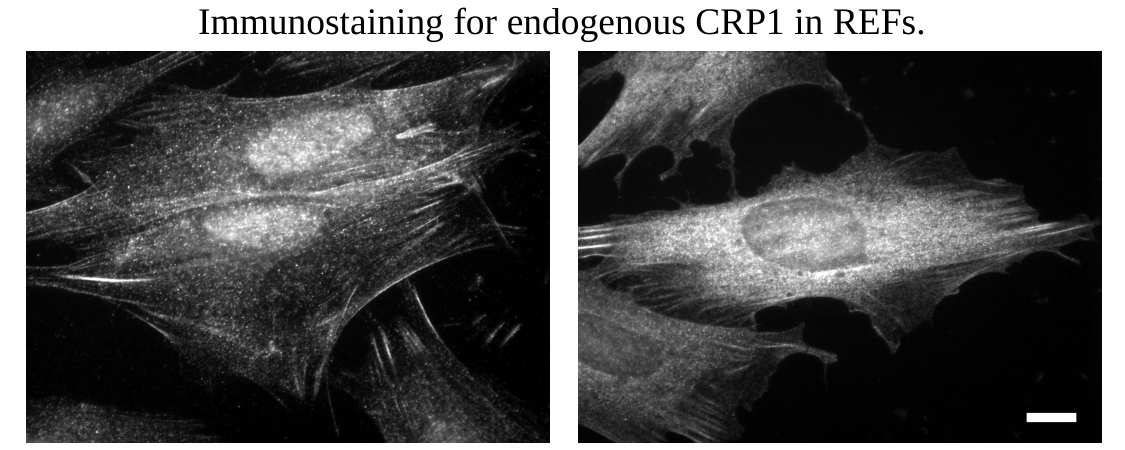

Immunostaining for endogenous CRP1 in REFs.

Supplement: Additional File 2 — Immunostaining for endogenous CRP1. Fluorescence microscopy of REFs stained with antibodies recognizing the C-terminal 17 amino acids residues (PKGFGFGQGAGALVHSE) of rat CRP1. Bar = 10 μm. [file 1471-2121-6-45-S2.ppt]
